# Andrew Lawrence, Editor‐in‐Chief 2018–2020

**DOI:** 10.1002/prp2.702

**Published:** 2020-12-10

**Authors:** Michael F. Jarvis, Jennifer Martin

Prof. Andrew Lawrence completes his tenure as the Editor‐in‐Chief of *Pharmacology Research & Perspectives (PR&P)* on December 31, 2020. Under Andy's guidance, the journal has experienced remarkable growth over the last several years. Submissions to the journal including papers cascaded from supporting journals and direct submissions have increased by 155% over the last 3 years. Importantly, *PR&P* received its first Impact Factor of 2.052 earlier this year and ranks in the top three of pharmacology and pharmacy journals. Andy has also overseen the development of several *PR&P* Special Issues that highlight scientific advances from early career researchers as well as key topics including advances in research methodology, negative findings, and current perspectives and reviews in key areas of pharmacology. Additionally, *PR&P* contributed to several Joint Special Issues from other British Pharmacology Society and American Society for Pharmacology and Experimental Therapeutics journals on pain, diabetes and sex as an experimental variable. Andy has led efforts to broaden the scope of the science published in *PR&P* as evidenced by the increased representation of clinical pharmacology and clinical outcomes research papers in the journal. He was also instrumental in expanding the geographic reach of *PR&P* with the establishment of regional associate editors and expanding the diversity of the editorial advisory board. Andy has also worked diligently to increase the quality and timeliness of the peer‐review feedback to the authors. A reflection of these efforts is the fact that the authors frequently send thank you notes to the editorial staff for the efficient and constructive feedback they have received.

Andy is a tireless champion of the journal. The *PR&P* editorial board and management committee thank him for his collegiality, mentorship, and many valuable contributions as *PR&P* continues to serve the pharmacology research community.

